# A Plea for Chloroform

**Published:** 1880-07-20

**Authors:** 


### A PLEA FOR CHLOROFORM.

A. G. Smythe, M.D., of Baldwyn, Miss., writes in the New York Medical Record:

Will you be so kind as to permit a backwoods general practitioner, to protest against the summary conviction and capital execution of chloroform as proposed in a recent number of the Medical Record by your Milwaukie, Wis. correspondent. While I am and have all my life been only a general practitioner, most of the time in the country, and cannot marshal surgical cases by the thousands, yet I propose to offer a few facts that have come within my own knowledge.

At the outset it will be admitted that chloroform is a potent agent, and should be used with the utmost caution and prudence, the routine description of which will be supposed to be well known to your readers.

Immediately after the publication of the success of the administration of chloroform by Sir James Y. Simpson (say as early as January, 1848), I procured a small quantity, with which I experimented upon myself in all the ways that I could without an assistant, and discovered that it almost invariably caused nausea and vomiting if taken a short time after eating. As early as February in the same year (1848) I administered it to a small boy, aged eight years, without any assistant or any person being present, and successfully performed a tedious and painful minor operation in the mouth, entirely free from pain or any untoward circumstances. (Will not give it in operations upon the nose, mouth or throat, at present—it is not safe.) From that day to the present time have used it—as all the therapeutic agents are used by myself, with prudence, paying but little attention to the host of so-called contraindications, except in the operations parenthesized—in a large number of capital, and a host of minor operations in surgery, also in general practice; in at least one thousand and four hundred, out of two thousand and six hundred cases of obstetries, without a failure in a single case. Only in four cases were there any symptoms to cause alarm on the part of those present, and but two that caused solicitude to your contributor, There is no agent in the whole materia medica with which your contributor has had such uniform success as that of chloroform, having at no time in thirty-two years failed to obtain all that was anticipated when it was given. No mortality (not a single case), where chloroform could be charged with any agency in causing a fatal result. There is no other remedy (not even quinine, and I have but few charges against it), which has been given to the same extent, that has given such entire satisfaction as the one in question, and now, at this late day, to presume to summarily dismiss a remedy which has passed through such an ordeal, and that can muster an array of friends and vouchers who, in point of character, skill and numbers, would strike consternation to the enemies of chloroform, the proposition as made by your contributor is, to say the least, summary and premature. Give chloroform and its friends a hearing; raise a committee or commission of investigation. The friends of chloroform do not fear a fair investigation.

In the future there may be discovered (I trust there will be) a safer and more pleasant anæsthetic than chloroform; but that it has been done up to the present time is doubted by a very large and respectable number of the profession throughout the civilized world.

Did time and space permit, much might be urged in this article as to the mode of administering chloroform, as compared with other remedies of the same class. Being more active in its action, it should be given slower, is all that will be said at present.

As an apology to your readers; will say that your contributor is by no means as young as he has been, and is at present almost blind; but was determined to come to the rescue of chloroform; not, however, that he had any fears that it would be dismissed from the Materia Medica.—*N. Y. Med. Rec.*

